# 

**Published:** 1908-11-14

**Authors:** 


### BOOKS RECEIVED.

John Murray.

" Therapeutics of the Circulation." By Sir Lauder Brunton, Bt., M.D.

" Letters of Queen Victoria, 1837-1861." Three vols.

" The Problem of Age, Growth, and Death." By Chas. S. Minot, D.Sc.

G. Routledge and Sons, Ltd.

" The Case for the Goat." By " Home Counties." Second edition.

Taylor and Francis.

" Third Scientific Report on the Investigations of the Imperial Cancer Research Fund." By Dr. E F. Bashford.

H. and W. Brown, Fulham Road, S.W.

" Glanders, a Clinical Treatise." By W. Hunting, F.R.C.V.S.

Hy. Frowde and Hodder and Stoughton.

" The Rectum, its Diseases and Developmental Defects." By Sir Charles Ball, M.Ch., F.R.C.S.I.

" Diseases of the Heart." By James Mackenzie, M.D., M.R.C.P

" Diseases of the Eye." By A. Stephen Mayou, F.R.C.S.

THE
### GRADUATES' MEDICAL SCHOOLS.

DIARY FOR WEEK NOV. 16 to NOV. 21.

### MEDICAL GRADUATES' COLLEGE AND POLYCLINIC, 22 Chenies Street, W.C.

**At 5.15 p.m.**

**Nov. 16,** Dr. Langdon Browne, **Nutrient Enemata and other Special Forms of Feeding.**

**Nov. 17,** Sir Felix Semon, **The Question of Pneumococcus Invasion of the Throat.**

**Nov. 18,** Dr. A. E. Giles, **The After Treatment of Abdominal Operations.**

**Nov. 19,** Dr. T. W. Eden, **The Coincident Occurrence of Certain Gynæcological Diseases.**

### LONDON SCHOOL OF CLINICAL MEDICINE, Seamen's Hospital, Greenwich, S.E.

**At 3.15 p.m.**

**Nov. 17,** Mr. Carless.

**At 2.15 p.m.**

**Nov. 20,** Dr. Rose Bradford, **Non-valvular Disease of the Heart.**

### ROYAL COLLEGE OF PHYSICIANS, Pall Mall East.

**Nov. 17 and 19,** Dr. F. W. Pavy, **The Pathology and Treatment of Diabetes Mellitus, viewed by the Light of Present Day Knowledge.**

### THE POST-GRADUATE COLLEGE, West London Hospital, Hammersmith, W.

**At 10 a.m.**

**Nov. 16 and 19,** Mr. Etherington Smith, **Demonstration of Surgical Cases.**

**At 12 noon.**

**Nov. 16,** Dr. Low, **Pathological Demonstration.**

**At 12.15 p.m.**

**Nov. 18 and 20,** Dr. Pritchard, **Practical Medicine.**

**At 5 p.m.**

**Nov. 16,** Mr. Baldwin, **Surgery, III.**

**Nov. 17,** Dr. Robert Jones, **The Physical Basis of Insanity.**

**Nov. 18,** Mr. Lloyd Williams, **Mouth Hygiene.**

**Nov. 19,** Mr. Edwards, **Urinary Cases.**

**Nov. 20,** Dr. Abraham, **Cases of Skin Disease.**

### ST. JOHN'S HOSPITAL FOR DISEASES OF THE SKIN, Leicester Square, W.C.

**At 6 p.m.**

**Nov. 19,** Chesterfield Lecture, **Tuberculosis of the Skin.**

### MOUNT VERNON HOSPITAL, Fitzroy Square.

**At 5 p.m.**

**Nov. 19,** Dr. George Johnston, **Prognosis in Phthisis.**

### THE HOSPITAL FOR SICK CHILDREN, Great Ormond Street, W.C.

**At 4 p.m.**

**Nov. 19,** Mr. Corner, **The Treatment of Some Cases of Talipes.**

### THE WEST-END HOSPITAL FOR NERVOUS DISEASES, Welbeck Street, W.

**At 5 p.m.**

**Nov. 19,** Dr. Dundas Grant, **Some Neuroses of the Throat, Nose, and Ear.**

### THE THROAT HOSPITAL, Golden Square, W.

**At 5.30 p.m.**

**Nov. 16,** Mr. Rose, **Disease of the Accessory Sinuses of the Nose: Symptoms and Diagnosis.**

**Nov. 19,** Mr. Parker, **Disease of the Accessory Sinuses of the Nose: Treatment.**

### NATIONAL HOSPITAL FOR THE PARALYSED AND EPILEPTIC, Queen Sq., Bloomsbury, W.C.

**At 3.30 p.m.**

**Nov. 17,** Dr. Ormerod, **Locomotor Ataxia.**

**Nov. 20,** Dr. Grainger Stewart, **Tumours of the Cauda Equina.**

### NORTH-EAST LONDON POST-GRADUATE COLLEGE, Prince of Wales's General Hospital, Tottenham, N.

**At 4.30 p.m.**

**Nov. 19,** Mr. J. H. Evans, **Symptoms Attributed to the Movable Kidney.**

### RECENT APPOINTMENTS.

Dr. W. E. Dixon, M.A., of Downing College, Professor of Pharmacology at King's College, London, and recently assistant to the Daning Professor of Medicine in the University of Cambridge, has been recommended for the post of University Lecturer in Pharmacology at Cambridge.

Dr. Joseph C. Muir has been appointed Medical Superintendent of the West Ham Infirmary, vice Dr. R. Niven, deceased. Dr. Muir is at present in charge of St. George's Infirmary and Children's Home at Fulham.

Dr. G. Thornton, Medical Superintendent of the Pretoria Hospital, Transvaal, has been appointed to succeed Dr. T. F. Garvin as Superintendent and Administrator of the General Hospital, Colombo, Ceylon. Dr. Thornton, before entering the service of the Transvaal Government in the year 1900, served for six years as Medical Officer in Metropolitan Asylums Board Fever Hospitals, and as Civil Surgeon attached to his Majesty's Forces in South Africa.

At the last meeting of the Council of the Pharmaceutical Society Miss Holland Wren was appointed demonstrator in the Society's School of Pharmacy. This is the first time a woman has been appointed to such a position since the school was established over 60 years ago. Miss Wren, it will be remembered, was recently awarded the Pereira Medal, and was the first woman to obtain that honour, which is the blue ribbon of pharmacy.

### ANSWERS TO CORRESPONDENTS.

W. Stead.—Your letter has been forwarded to the author of the article in question.

THE HOSPITAL November 14, 1908.

*Name* ..............................................................

*Address* ..............................................................

..............................................................

This Coupon must accompany manuscript or contributions intended for The Hospital.

